# Caregiver Emotional Burden in Testicular Cancer Patients: From Patient to Caregiver Support

**DOI:** 10.3389/fendo.2019.00318

**Published:** 2019-05-28

**Authors:** Silvia De Padova, Chiara Casadei, Alejandra Berardi, Tatiana Bertelli, Alessia Filograna, Maria Concetta Cursano, Cecilia Menna, Salvatore Luca Burgio, Amelia Altavilla, Giuseppe Schepisi, Sabrina Prati, Sandra Montalti, Michal Chovanec, Giuseppe Luigi Banna, Luigi Grassi, Michal Mego, Ugo De Giorgi

**Affiliations:** ^1^Psycho-Oncology Unit, Istituto Scientifico Romagnolo per lo Studio e la Cura dei Tumori (IRST), IRCCS, Meldola, Italy; ^2^Medical Oncology Department, Istituto Scientifico Romagnolo per lo Studio e la Cura dei Tumori (IRST), IRCCS, Meldola, Italy; ^3^Medical Oncology Department, Campus Bio-Medico University, Rome, Italy; ^4^2nd Department of Oncology, Faculty of Medicine, Comenius University and National Cancer Institute, Bratislava, Slovakia; ^5^Division of Medical Oncology, Cannizzaro Hospital, Catania, Italy; ^6^University Hospital Psychiatry Unit, Integrated Department of Mental Health and Addictive Behavior, St. Anna University Hospital and NHS Community Health Trusts, Ferrara, Italy

**Keywords:** caregiver, testicular, cancer, patients, long-term survivors

## Abstract

Testicular cancer is the most common tumor in young males aged 15–40 years. The overall cure rate for men with testicular cancer is >90%, so a huge number of these patients will become testicular cancer survivors. These people may feel some stress in the experience of diagnosis, treatment, and consequences that affects the quality of life, and during follow-up, especially when new issues and emotional distresses appear over time, such as late side-effects of treatments and emotional challenges including fear of tumor relapse, fertility and sexuality concerns, and social and workplace issues. The cancer experience has an impact not only on patients, but also on their relatives (e.g., spouses, parents, or siblings), who often have to assume a caregiving role for the duration of and following treatment for cancer. Moreover, the caregiver plays an important role in supporting a man with a testicular cancer, providing physical and emotional care. This review presents a summary of existing knowledge regarding the impact and the burden of testicular cancer on caregivers.

**Graphical Abstract:**
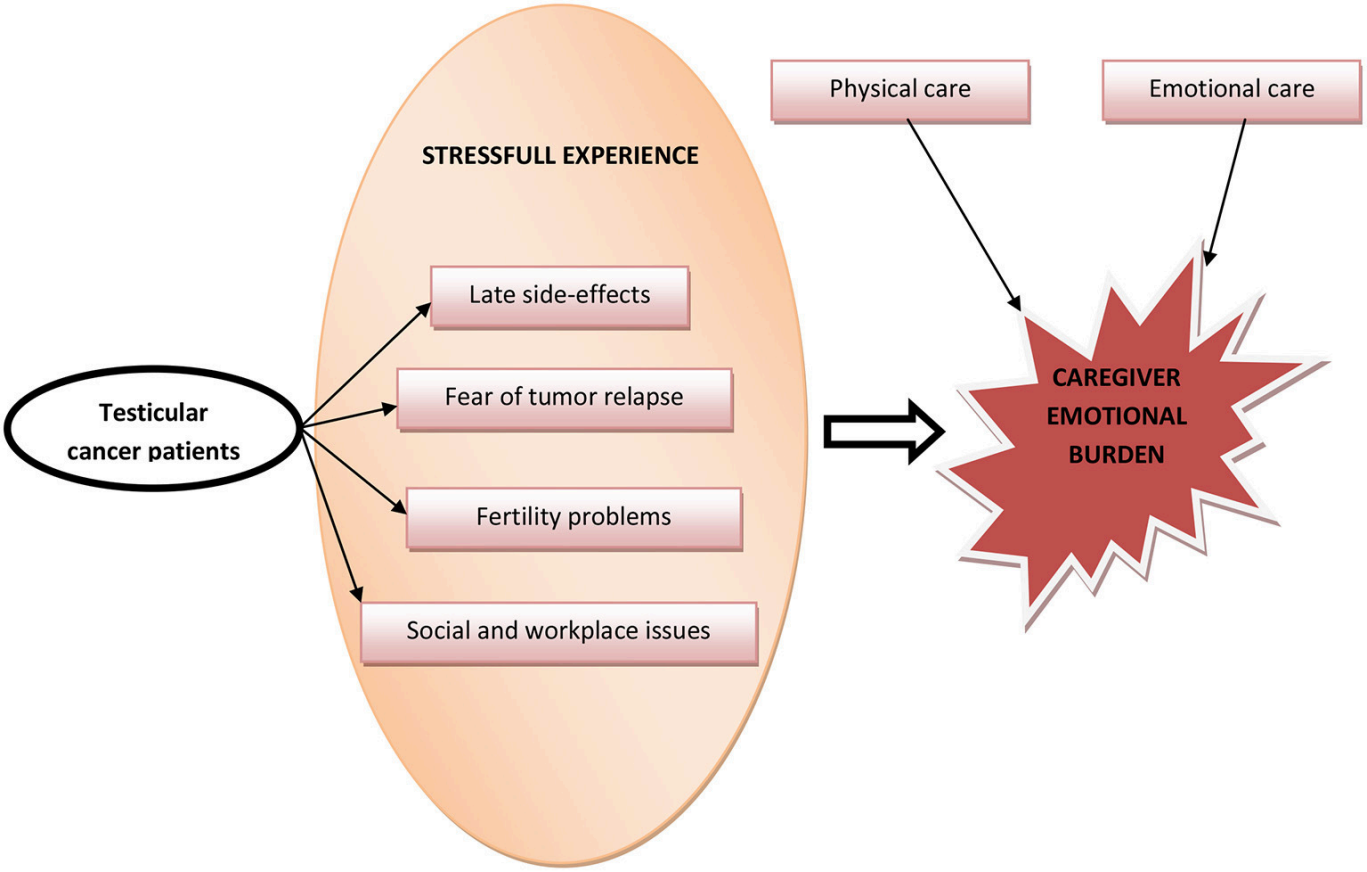
Factors influencing Caregiver emotional burden in TC patients.

## Introduction

Testicular cancer (TC) is the most frequent solid tumor in young adult men aged between 15 and 35 years, and is a highly curable cancer with survival rates close to 99% for stage I disease cases (localized tumor) and 80–90% for cases with metastatic disease treated with cisplatin-based chemotherapy and surgery on residual masses, when indicated (1). In addition, the 10–20% of metastatic patients who are not cured with first-line cisplatin-based chemotherapy, increase their chances of long-term remission in nearly 50% of cases treated with second-line treatments, such as high-dose chemotherapy (HDCT) or standard-dose chemotherapeutic regimens, and in nearly 15–30% of cases treated in the following lines with other salvage regimens (2–5). A young age at diagnosis and excellent prognosis, physical, psychological and social well-being represent a significant indicator for follow-up and survivorship of these people. In fact, despite the excellent prognosis, cured patients may experience long-term somatic sequelae and psychosocial distress according to the tumor and treatment burden (6, 7). However, type and duration of each treatment depends on initial stage of the disease (Table 1). As a consequence, different physical and psychological loads correlate with different treatment loads. Both tumor diagnosis and tumor treatment are usually stressful events affecting not only patients but the whole family system (Graphical Abstract) (8).

**Table 1:** Therapeutic strategies, clinical complications, and correlation with QoL.

**Histology**	**Stage**	**Primary treatment**	**Most common adverse events**	**Detrimental effect on QoL**	**Stage**	**Secondary treatment**	**Most common adverse events**	**Detrimental effect on QoL**
Seminoma	I	Single agent Carboplatin (AUC7 for 1 cycle) or RT (20 or 25 Gy)	Myelotoxicity Fatigue	Low risk	Relapsed/Refractory TC	VeIP (4 cycles) or TIP (4 cycles) or HDCT ± RT	Myelotoxycity Fatigue Alopecia Vomiting Neurotoxicity Infertility Cardiovascular toxicity Pulmonary toxicity Solid secondary tumors Leukemia	High risk
	IIA	RT (30 Gy)	Fibrosis	Low risk				
	IIB	BEP (3 cycles) or EP (4 cycles) or RT in selected non-bulky cases	Myelotoxycity Fatigue Alopecia Vomiting Neurotoxicity Infertility Pulmonary toxicity Solid secondary tumors Leukemia	Intermediate risk				
	IIC, III	BEP (3 or 4 cycles)						
Non-seminoma	I	Surveillance or BEP (1 cycle) or RPLND	If BEP: Myelotoxicity Fatigue Vomiting Alopecia	Intermediate risk				
	II, IIIA	BEP (3 cycles) or EP (4 cycles) or Nerve-sparing RPLND	Myelotoxycity Fatigue Alopecia Vomiting Neurotoxicity Infertility Pulmonary toxicity Solid secondary tumors Leukemia	Intermediate risk				
	IIIB, IIIC	BEP (4 cycles) or VIP (4 cycles)						

*RT, radiotherapy; BEP, Bleomycin, Etoposide, and Cisplatin; EP, Etoposide and Cisplatin; RPLND, retroperitoneal lymph-node dissection; VIP, Etoposide, Ifosfamide, and Cisplatin; VeIP, VeIP Vinblastine, Ifosfamide, Cisplatin; TIP, Paclitaxel, Ifosfamide, and Cisplatin; HDCT, high dose chemotherapy. Stage and primary treatment according to NCCN guidelines version 1.2019^1^*.

### Role of Caregiver

Caregivers are individuals, usually family members or relatives (e.g., partner, parents, but also close friends), who have a significant relationship with the patient suffering from a life-threatening illness and provide assistance (9, 10). Along the whole process of the disease and its treatments, they are engaged in the practical help and psychological support for coping with the situation, including the emotions of uncertainty and fear (11). This role requires many abilities that may be physically, emotionally and financially demanding. The burden of caregiving has been defined a “multidimensional biopsychosocial reaction resulting from an imbalance of care demands relative to caregivers' personal time, social roles, physical and emotional states, financial resources, and formal care resources given the other multiple roles they fulfill” (12).

Cancer could determine major effects both on caregivers and patients, with literature clearly indicating that cancer affects the emotional, social, physical, and spiritual well-being of patients and their family members (13).

Most studies in family caregivers of cancer patients reported diverse problems as a consequence of their role, ranging from diminished physical health and psychological distress to an adverse impact on their work (14). The most prevalent physical problems included sleep disturbance, fatigue, pain, loss of physical strength, loss of appetite, and weight loss.

Cancer is a family experience that exerts a change in family's system, balance and identity, redefines the rules, changes the lives of all its members, brings an immense amount of stress, and presents many challenging situations. Cancer and the approaches used to treat it can introduce a complex array of lifestyle changes and emotional responses, which can be difficult for family members to handle. The diagnosis of cancer, its treatment and symptoms both of the illness and of the chemotherapy have an influence on how patients and their caregivers experience distress. A review on psychological impact of cancer on patients' partners and other relatives affirmed that an important minority of carers become highly distressed, clinically depressed and anxious: in particular, prevalence of clinically significant distress among caregivers was reported to be 20–30% in studies using self-report questionnaires, whereas in studies that used diagnostic interviews rates are approximately 10% (15). However, data concerning caregivers' distress are sparse. This review analyzes TC-related distress and burden of caregivers.

## Caregiver Burden

The experience of illness perceived by the caregiver depends on some specific aspects of the disease such as the type of cancer and the stage of life in which it is diagnosed.

TC has a profound effect on body image and on the personal image of oneself and, and often occurs in adolescence and young adulthood, times characterized by significant life changes and psychosocial challenges. These men are in the prime of their lives, when health is often taken for granted, while interpersonal relationships and the desire to start a family may be major interests (16–19).

Diagnosis of TC causes a sort of emotional earthquake which involves relatives, partners and close friends. Cancer patients during treatment and also during the follow-up period experience many needs, and caregivers are often unprepared to respond to this important burden (20). Main psychological problems experienced by TC caregivers are summarized in Table 2. Of note is that the physical and mental health of patients and their caregivers are often related. In this contest, improving knowledge and social support to caregivers could help to ameliorate patients' global health. Confirming this, depression symptoms are less frequent in patients living in couples (21). Taking care of a patient with cancer is described as a full-time job (22) and caregivers themselves often are in great need of psychological support. Caregivers of TC patients usually are parents or partners, typically young females and it is recognized that the highest predisposition to symptoms of distress have been showed by female caregivers of young age and lower social status (19). Therefore, for female partners of cancer patients, there is a risk of developing psychological and psychiatric morbidity and lower quality-of-life (QoL) than women in healthy couples (15, 23). In this contest, TC survivors' caregivers are a high risk population. Several studies reported rates of anxiety of between 30 and 50% (24) among family caregivers in comparison to rates of anxiety of between 19 and 34% (25) in patient samples. Likewise, rates of depression are reported to be between 10 and 25% (25) in patients samples compared to between 12 and 59% (24) among family caregivers. This underlines that, in many cases, the psychological burden on caregivers is even greater than in patients (Table 3). Caregivers receive less practical and emotional support from friends and professionals than patients (26). However, their high self-efficacy can improve their own mental state and also the mental well-being of patients.

**Table 2:** Main psychological issues in TC caregivers.

**Main psychological problems in TC caregivers**
Need of information
Anxiety
Depression
Inadequacy
Lack of practical and emotional support
Risk of infertility
Sexual difficulties

**Table 3:** Differences in emotional burden between TC patients and their caregivers.

	**Caregivers (%)**	**Patients (%)**
Anxiety	30–50 (24)	19–34 (25)
Depression	12–59 (24)	10–25 (25)

In their caregiver role, partners of TC patients have a social task: commonly they give information to family members and friends while her husband is in the hospital (27). Another considerable aspect of these women's burden is emotional experiences connected to the illness and to the period of life in which it occurs. Often the diagnosis of TC happens in an important period of life, characterized by major life changes and specific developmental tasks, when forming intimate and long-term emotional and sexual relationships, and starting families may be major concerns (28). Each member of the couple, both the patient and the partner, is faced with the possibility of treatment-related infertility and sexual difficulties in a period of life where partners are often focused on starting a family (27). However, only a minority of the couples experienced more serious and long lasting testicular cancer-induced disturbances in sexual and marital relationships. In general, couples felt their relationship became more tightly bonded and stronger following the confrontation with TC (29, 30).

Both psychosocial and QoL consequences occur years after the experience of the tumor and the end of treatments. Tuinman et al. (31) showed that spouses who experienced the diagnosis and treatment process had better physical QoL than the average woman. Their stress response levels were low and were related to the stress response level of TC survivors and to the duration of treatments received. However, these women, even years after the completion of treatment, were experiencing more stress response symptoms than the TC survivors.

Relevant components of the caregiver's burden consist of the support, assistance and information needs that, if not unmet, leads to reduced QoL and high levels of distress (32). Kim et al. (33) demonstrated that caregivers of cancer patients frequently have a variety of unmet needs and that unmet needs strongly predicted their QoL.

Patients, caregivers and care providers had different expectations about TC survivorship: psychological distress was considered as highly relevant by 35% of patients and caregivers and 93% of care providers; the couple's relationship was quite or very difficult for 12% of patients and caregivers in comparison to 64% in the perception of care providers (34). A different perception of the illness experience could affect the recognition and ability to respond to the needs of patients and caregivers. In another study, close relatives of men suffering from TC highlight four themes: the disease and its course, normalization, the long-term consequences, and the social network (35). The results showed that relatives suffer from social isolation (35).

Another source of caregiver burden could be the fertility issue: the paternity rates among men who attempted to conceive a child after treatment were 71% at 15 years and 76% at 20 years after orchiectomy, but this rate ranged from 48% in the HDCT group to 92% in the surveillance group (36). Sandén and Söderhamn (37) reported a conversational interview to a young woman whose partner had TC using a semistructured guide with open-ended questions. Caring became primary for female partner, and she focused less on her own needs in order to support the patient; everyday life changed, as more time was spent at the hospital, the home, and the parent's home. The third keypoint was the shortness of time: from the discovery of the disease and the start of chemotherapy, time was reported as passing very quickly, and the felt like they spent a lot of time with their physicians.

In literature, little data exists about the role of mothers as caregiver of TC patients and the dramatic changes in their lives. Unlike their healthy peers, young TC patients often face greater challenges in life: they may experience delays in developmental milestones, difficulties in employment and interpersonal relationships, and medical and institutional problems (e.g., economy, education, transport). These challenges can hinder their transition to independence, which is not favored by mothers who continue to take care even when their sons progress to adulthood (38).

## Caregiver Therapy

Caregivers need a large volume of information, including: diagnosis-related information, prognosis-related information, treatment-related information, information on homecare, and information about impact on the family or on relationship with partner. Therefore, psychoeducational interventions have been conceived to increase their knowledge. Bultz et al. (39) reported that this sort of intervention had a positive impact on caregivers' ability to provide care and also improve marital satisfaction of patients. Pelusi et al. (40) revealed that caregivers sharing their cancer experience with others in storytelling is essential to offer educational information and emotional support to those who hear it, but also care for self is an important component of managing the course of these events. A Chinese study explored the relationship between family resilience and the post-traumatic growth, and the quality of life of survivors of breast cancer, demonstrating that family resilience decreased caregivers' and patients' burden (41). One intervention used telephone interpersonal counseling, which was delivered to patients and their caregivers separately to improve cancer education and resulted in significant decreases in depression and anxiety levels in the caregivers group (42). Kozachik et al. (43) conducted a quasi-experimental study to describe the use of complementary therapy (such as reflexology, guided imagery, and reminiscence therapy) to cancer patients undergoing chemotherapy and their family caregivers. The authors were unable to draw conclusions regarding the impact of complementary therapy on caregiver burden, however they suggest that one complementary therapy may be incorporated into patients' and caregivers' courses of cancer treatment (43).

According to the different stage of the disease at diagnosis, several treatment strategies are recommended (Table 1). These modalities are associated with different complications and late toxicities and a negative impact on QoL. TC survivors have a high risk of leukemia; the relative risk, associated with the previous use of etoposide, ranges between 3.5 and 4.5 and appears often within 10 years following the end of treatment (44). Younger age at radiotherapy and/or chemotherapy increases risk for solid secondary tumors and remains elevated for at least 35 years (45). In long-term setting pulmonary toxicity, infections and cardiovascular events are higher compared with the general population (46, 47). For TC survivors and their caregivers, preserved fertility is a fundamental subject which has an important impact on their QoL (48). The prospect of paternity improves with the decreasing number of cycles of chemotherapy, therefore the correct management of TC requires a careful balance between the intensity of treatment and burden of disease, in order to limit short and long term adverse events (49). In sight of this, the correct management of late toxicities is essential in order to preserve the higher QoL of patients and their caregivers. A recent multidisciplinary consensus conference by the Italian Germ cell cancer Group (IGG) and the Associazione Italiana di Oncologia Medica (AIOM) has provided recommendations for surveillance and follow-up appointments of men with TC, suggesting a visit with caregiver at the beginning of follow-up and, eventually, a psychological consultation (50).

To date there are no evidence about the importance of the role of nurse in supporting caregivers of TC-patients. In our opinion, it is important to develop educational programs with the aim of creating a cancer clinical nurse specialist role, who could support patients and their families during the course of disease. This program could guarantee personalized nursing assistance and aid (both psychological and practical), leaving from the psychosocial contest of the patients. The role of nurse is expressed before, during and after the identification and the monitoring of signs and symptoms of the disease and the treatment, in order to promote the well-being of the patients and their caregivers.

TC patients and their families have to be included; they should have the opportunity to be involved in the planning of the assistance and in the decision making process through individualized services which could adapt to the changing psychophysical status of the patients (51).

## Conclusions

Cancer treatment is not an individual experience, but induces deep effects on patients' families. Couples who achieve the survivorship phase often have to change life plans, deal with TC and treatment-related effects, and manage worries about future health (52). Patients partnered at diagnosis experience a better emotional and physical adaptation to disease (53, 54) and the majority of follow-up studies reported that the rate of divorce or broken relationship was 5% to 10% (55). The impact of disease on caregivers depends also on patients' story. TC patients who undergo, after orchiectomy, one cycle of chemotherapy probably have a lower burden of distress compared with patients who complete four standard cycles of chemotherapy, due to the reduced treatment load. Moreover, some patients are not cured with first-line chemotherapy and have to be treated with further intensified chemotherapeutic regimens, including standard dose chemotherapy supported by granulocyte-colony stimulating factor (G-CSF) (3) and/or HDCT with support of autologous peripheral-blood stem-cell (56, 57). HDCT is a stressful program both for patients and their family, due to long hospitalization periods and a high risk of treatment-related toxicity that requires the adoption of specific precautionary measures. These therapeutic options are able to lead to long-term remission of disease, but leave a stressful emotional burden on the patient and his caregivers. There is some data available about stressful burden of caregivers of elderly persons with physical dependence (58), but there are little evidence about young patients' caregivers, though often they are young people at risk of psychological distress which could have an impact on long-term effects (59, 60).

Unfortunately, there are little data available in literature concerning the role of caregiver in TC patients, probably because of the lower number of persons involved compared with breast cancer, for example. However, in our opinion, according to the young age of patients and the very good prognosis, it is important to consider a more integrated system of the patient and his social support, with the purpose of improving QoL not only during active treatment, but also in the follow-up period, and to encourage a less traumatic return to the everyday life.

## Author Contributions

SD, CC, and UD collaborated in the conception, in the data retrieval, and in the drafting of the text. CC, GS, and MC collaborated in the revision of the text and in the completion of the bibliographic research. AB, TB, AF, CM, SB, AA, SP, SM, MC, GB, LG, and MM revised the manuscript.

### Conflict of Interest Statement

The authors declare that the research was conducted in the absence of any commercial or financial relationships that could be construed as a potential conflict of interest.

## Footnotes

^1^https://www.nccn.org/professionals/physician_gls/pdf/testicular.pdf
